# Novel Food and Beverages: Production and Characterization

**DOI:** 10.3390/foods13132085

**Published:** 2024-07-01

**Authors:** Víctor M. Perez-Puyana, Mercedes Jiménez-Rosado, Alberto Romero

**Affiliations:** 1 Departamento de Ingeniería Química, Universidad de Sevilla, 41012 Sevilla, Spain; alromero@us.es; 2 Department of Applied Chemistry and Physics, Universidad de León, 24007 León, Spain; mjimr@unileon.es

## 1. Introduction

In today’s market, the pursuit of product optimization is not just a trend but a necessity while facing the challenges of supplying a wider range of products, sustainability pressures, and the requirement for continuous innovation [1,2,3].

The situation at present is prompting researchers and manufacturers in the food and beverage industry to focus on development and optimization [4,5,6,7]. These aspects are approached from several perspectives. From enhancing the nutritional properties of food products to the incorporation of new food sources, studies are being conducted to optimize the manufacturing process [2,8]. This involves improving overall production performance by optimizing processing techniques or improving product storage [9,10,11].

The following Editorial refers to the Special Issue “Novel Food and Beverages: Production and Characterization”. The Special Issue emphasizes the evaluation of novel food from a rheological point of view, with the included studies discussing novel and traditional characterization techniques from the perspectives of rheology, food safety, sustainability, process engineering, (bio)chemical changes, and sensory issues.

## 2. Overview of the Published Articles

Nine manuscripts were submitted for the aforementioned Special Issue, and they were all subject to the journal’s rigorous review process. In total, seven papers were ultimately accepted for publication and inclusion in this Special Issue. The contributions are listed below:

A. Wang, H.; Yuan, J.; Chen, L.; Ban, Z.; Zheng, Y.; Jiang, Y.; Jiang, Y.; Li, X. Effects of Fruit Storage Temperature and Time on Cloud Stability of Not from Concentrated Apple Juice. *Foods* **2022**, *11*, 2568. https://doi.org/10.3390/foods11172568.
B. Ahmed, T.; Sabuz, A.A.; Mohaldar, A.; Fardows, H.M.S.; Inbaraj, B.S.; Sharma, M.; Rana, M.R.; Sridhar, K. Development of Novel Whey-Mango Based Mixed Beverage: Effect of Storage on Physicochemical, Microbiological, and Sensory Analysis. *Foods* **2023**, *12*, 237. https://doi.org/10.3390/foods12020237.
C. Torrey, K.J.; Liu, Y.; Li, H.; Ma, H.; Via, C.W.; Bertin, M.J.; Seeram, N.P. What Is Authentic Maple Water? A Twelve-Month Shelf-Life Study of the Chemical Composition of Maple Water and Its Biological Activities. *Foods* **2023**, *12*, 239. https://doi.org/10.3390/foods12020239.
D. Simões, S.; Carrera Sanchez, C.; Santos, A.J.; Figueira, D.; Prista, C.; Raymundo, A. Impact of Grass Pea Sweet Miso Incorporation in Vegan Emulsions: Rheological, Nutritional and Bioactive Properties. *Foods* **2023**, *12*, 1362. https://doi.org/10.3390/foods12071362.
E. Liu, R.; Yang, Y.; Cui, X.; Mwabulili, F.; Xie, Y. Effects of Baking and Frying on the Protein Oxidation of Wheat Dough. *Foods* **2023**, *12*, 4479. https://doi.org/10.3390/foods12244479.
F. Jafari, S.; Shiekh, K.A.; Mishra, D.K.; Kijpatanasilp, I.; Assatarakul, K. Combined Effects of Clarifying Agents Improve Physicochemical, Microbial and Sensorial Qualities of Fresh Indian Gooseberry (*Phyllanthus emblica* L.) Juice during Refrigerated Storage. *Foods* **2024**, *13*, 290. https://doi.org/10.3390/foods13020290.
G. Kim, S.-Y.; Lee, Y.-G.; Ju, H.-I.; Jeon, J.-H.; Jeong, S.-H.; Lee, D.-U. The Effects of Jet-Milling and Pulsed Electric Fields on the Preservation of Spinach Juice Lutein Contents during Storage. *Foods* **2024**, *13*, 834. https://doi.org/10.3390/foods13060834.

Wang et al. (A) evaluated the influence of different storage conditions on the properties of apple juice. Specifically, different temperatures and storage times were analyzed to optimize the extraction of juice in water-cored Fuji apples. Regarding the study results, longer storage periods were found to be necessary when the storage temperature decreases, as shown by the best juice stability being obtained at 45 days in 0 °C conditions. Conversely, when working at 20 °C, the time required was demonstrated to be significantly lower (15 days). As the main conclusion of the study, the stability of apple juices can be controlled by modifying the physiological state of the raw materials used.

Ahmed et al. (B) focused on the production of beverages based on the use of whey and mango. Different formulations were produced, and their acidity, chemical composition, sedimentation rate, and total plate count were analyzed. The main findings of this study revealed that whey-based products can be obtained using fruits and vegetables without significantly changing their properties in addition to sensory analysis results.

In contrast, Torrey et al. (C) evidenced the lack of a proper identity for maple water products on the market because of the absence of standards of identity in the beverage market, leading to deficiencies in consumers’ knowledge of real maple water. For this reason, they studied the chemical composition of pasteurized maple water over its 12-month shelf life. Furthermore, the properties of a maple water extract and a chemically characterized phenolic-enriched maple syrup extract were compared. No significant differences were found in the physicochemical profiles and antioxidant capacity of both extracts. The study findings can aid in promoting the maple industry in this market sector.

Simoes et al. (D) focused on a multidisciplinary approach involving microbiological, rheological, and chemical methods to develop a new vegan emulsion with potential added value. The main raw material used was grass pea sweet miso. Different formulations were produced, with a specific formulation (7.5 wt.%) with adequate rheological properties, texture profile, and good stability being obtained. Furthermore, the final product showed a significant increase in phenolic content and antioxidant activity in comparison with the standard vegan emulsion.

Liu et al. (E) investigated the influence of protein oxidation on wheat dough during baking or frying. The amino acid composition, secondary structure of the proteins, and free radical production were evaluated. The findings of their study revealed that excessive baking and frying can cause protein oxidation and denaturation, decreasing nutritional value and digestibility and increasing the content of amino acid oxidation products. In this sense, their main conclusion confirmed that reducing excessive processing is essential in decreasing the negative effects of protein oxidation in food.

Jafari et al. (F) used gelatin and bentonite as potential clarifying agents to evaluate the potential improvement of the quality and shelf life of gooseberry juice. Different combinations were prepared, and the sensory quality, bioactivity, and color properties of the juices were analyzed. A specific combination of gelatin and bentonite was found to be the most effective formulation to preserve their quality and color properties. Moreover, the treated juices showed no signs of bacteria, yeast, or mold upon cold storage. Therefore, through their study, Jafari et al. were able to confirm that gelatin and bentonite could be the preferred filtering aids for quality and shelf-life extension in the food industry.

Kim et al. (G) studied the effect of jet-milling on the properties of extracted spinach powder. Furthermore, a pulse electric field was applied as a form of pasteurization technology to improve the preservation of spinach-based juice. Different particle sizes were analyzed during a specified storage period, with more homogeneous and smoother particles being exhibited when decreasing their size. In this sense, decreasing their particle size led to the highest lutein content and antioxidant activity.

## 3. Conclusions and Future Perspectives

Maintaining a healthy diet remains a global challenge in meeting the 2030 sustainable development agenda. As the human population continues to grow, researchers face increasing challenges to ensure that people around the world will have access to nutritious, safe, and healthy food. By 2050, the global human population is expected to reach almost 10 billion; therefore, food production will need to increase by more than 50% of 2012 production levels to meet demand. In this sense, an alternative to natural products is required, highlighting the environment as a significant potential source that has been scarcely examined. In light of the above, several approaches could be developed:

- The identification and characterization of new ingredients obtained from unexplored natural sources, i.e., animals, plants, or minerals sources not being currently used for human consumption. This should also encompass a detailed examination of the potential risks associated with the emergence of novel pathogens and foodborne diseases as a consequence of the incorporation of these new ingredients.
- The development of suitable, affordable, and environmentally friendly technologies for the extraction of food ingredients.
- The incorporation of new food sources into innovative food products and supplements and their development and commercialization.
- Life cycle analysis of food sustainability based on new consumer demand.

## References

[1] Giacalone D., Jaeger S.R. (2023). Consumer Acceptance of Novel Sustainable Food Technologies: A Multi-Country Survey. J. Clean. Prod..

[2] Guo X., Huang J., Wan X. (2024). Influence of Exposure to Novel Food Packaging on Consumers’ Adoption of Innovative Products. Food Qual. Prefer..

[3] Johnson S.L., Moding K.J., Flesher A., Boenig R., Campain J. (2024). I’ll Never Give Up: A Qualitative Study of Caregivers’ Perceptions and Decisional Processes When Feeding Infants and Toddlers Novel and Disliked Foods. J. Nutr. Educ. Behav..

[4] Mazac R., Järviö N., Tuomisto H.L. (2023). Environmental and Nutritional Life Cycle Assessment of Novel Foods in Meals as Transformative Food for the Future. Sci. Total Environ..

[5] Monaco A., Kotz J., Al Masri M., Allmeta A., Purnhagen K.P., König L.M. (2024). Consumers’ Perception of Novel Foods and the Impact of Heuristics and Biases: A Systematic Review. Appetite.

[6] Spatola G., Giusti A., Mancini S., Tinacci L., Nuvoloni R., Fratini F., Di Iacovo F., Armani A. (2024). Assessment of the Information to Consumers on Insects-Based Products (Novel Food) Sold by e-Commerce in the Light of the EU Legislation: When Labelling Compliance Becomes a Matter of Accuracy. Food Control.

[7] Tiefenbacher K.F. (2019). New Products Require New Thinking—Ideas and Examples. The Technology of Wafers and Waffles II.

[8] Huang M., Adhikari B., Lv W., Xu J. (2024). Application of Novel-Assisted Radio Frequency Technology to Improve Ready-to-Eat Foods Quality: A Critical Review. Food Biosci..

[9] Hendrich S. (2016). Novel Foods. Encyclopedia of Food and Health.

[10] de Boer A. (2024). Food Safety Requirements for Novel Foods. Encyclopedia of Food Safety.

[11] Guo Q., Zhang M., Mujumdar A.S., Yu D. (2024). Drying Technologies of Novel Food Resources for Future Foods: Progress, Challenges and Application Prospects. Food Biosci..

